# Real-Time Monitoring and Quantitative Evaluation of Resin In-Filtrant Repairing Enamel White Spot Lesions Based on Optical Coherence Tomography

**DOI:** 10.3390/diagnostics11112046

**Published:** 2021-11-04

**Authors:** Sujuan Zeng, Yuhang Huang, Wenyan Huang, Janak L. Pathak, Yanbing He, Weijian Gao, Jing Huang, Yiqing Zhang, Jian Zhang, Huixian Dong

**Affiliations:** 1 Department of Pediatric Dentistry, Affiliated Stomatology Hospital of Guangzhou Medical University, Guangzhou Key Laboratory of Basic and Applied Research of Regenerative Medicine, Guangzhou 510182, China; 2008690246@gzhmu.edu.cn (S.Z.); Huang-yuhang@foxmail.com (Y.H.); 2019686041@gzhmu.edu.cn (W.H.); j.pathak@gzhmu.edu.cn (J.L.P.); 2019217936@stu.gzhmu.edu.cn (Y.H.); 2 Department of Biomedical Engineering, School of Basic Medical Sciences, The Sixth Affiliated Hospital of Guangzhou Medical University, Qingyuan People’s Hospital, Guangzhou Medical University, Guangzhou 511436, China; wgaoweijian@gmail.com (W.G.); 2018161033@stu.gzhmu.edu.cn (J.H.); 2019161047@stu.gzhmu.edu.cn (Y.Z.); 3 Department of Endodontics, Affiliated Stomatology Hospital of Guangzhou Medical University, Guangzhou Key Laboratory of Basic and Applied Research of Regenerative Medicine, Guangzhou 510182, China; dhuixian@foxmail.com

**Keywords:** enamel, OCT, resin infiltrant, white spot lesions

## Abstract

The aim of the present study was to explore the feasibility of real-time monitoring and quantitative guiding the repair of enamel white spot lesions (WSLs) with resin infiltration by optical coherence tomography (OCT). Seven New Zealand rabbits were treated with 37% phosphoric acid etchant for 15 min to establish the model of enamel demineralization chalk spots of upper incisors, which were repaired by Icon resin infiltrant. OCT, stereo microscope (SM) imaging, scanning electron microscope (SEM) imaging and hematoxylin eosin (HE) staining were used to image each operation step. The changes of WSLs of enamel before and in the process of restoration with resin infiltrant showed specific performance in OCT images, which were consistent with the corresponding results of stereomicroscope and SEM. OCT can non-invasively and accurately image the whole process of repairing enamel demineralization layer with resin infiltration real-time, which can effectively guide the clinical use of resin infiltrant to repair enamel WSLs and be used as an imaging tool to evaluate the process and effect of restoration with resin infiltrant at the same time.

## 1. Introduction

Orthodontic brackets or plaque accumulation demineralize the tooth surface, leaving white spot lesions (WSLs) [1,2,3,4,5]. As an early and reversible form of dental caries, WSLs negatively affect the aesthetics and dental appearance of the tooth [6]. Current clinical treatment for WSLs includes fluoride remineralization, bleaching, microabrasion, and resin infiltration [5,7]. The resin infiltrant enters the micropores of the demineralized area through capillary infiltration and seals the micropores after combining with the tooth tissue. Resin infiltration prevents the acid from further diffusion, covering the lesions, and thus improving the smoothness and aesthetics of the tooth surface [8,9,10].

As a new method for minimally invasive repair of enamel white spot lesions [11], resin infiltrant incorporates into the porous space under the surface of demineralized enamel through the capillary siphon and seals the latter after polymerization [12]. Resin infiltration has a better effect on delaying and preventing enamel demineralization progress than traditional treatment methods such as fluorine coating [13]. However, the resin infiltrant cannot completely infiltrate into all the tiny pores [14,15]. After being repaired with resin infiltrant, there is still a demineralized area in the deep layer of the dental tissue. An in vitro experiment conducted by Andrej et al. [16] on the effect of resin infiltrant on the caries of extracted human premolars showed 50% pores without resin infiltrant. Meanwhile, earlier studies have demonstrated that bacterial colonization can be found in the enamel even when the enamel surface is intact in the early stage of enamel demineralization [17]. No definite conclusions have been made regarding the effect of bacteria in the uninfiltrated area on the restorative effect and lesion progression, and further studies are required.

However, at present, due to the limitation of imaging resolution (mm), cone-beam CT (CBCT) and other types of imaging equipment commonly used in the clinic are unable to distinguish abnormal enamel white spot lesions from normal dental tissues. At the same time, due to the high transmissivity of resin infiltrant, the above two imaging methods cannot visualize the image of the materials, nor can distinguish the restored tooth tissue from the unrepaired demineralized tissue and penetration depth of the resin. Therefore, the clinical evaluation of the effect of resin infiltrant restoration is still limited to the morphology and color changes of the tooth surface before and after restoration [18]. The authors did not find the inspection method to evaluate the infiltration effect of resin infiltrant.

In the 1990s, Huang et al. [19] proposed optical coherence tomography technology, which is based on the principle of low coherence interference to obtain depth tomography images. The light from the sample arm irradiates the object and then is refracted and scattered to produce coherent signals. The spectrometer collects coherent signals and sends the coherent signals to the computer. By reconstructing the profile of optical coherence between the light from the reference arm and the sample arm, OCT can obtain the two-dimensional or three-dimensional image of the sample [20,21,22,23]. The spatial resolution of the OCT image is about 10 μm. At present, the application of OCT mainly focuses on the imaging of eyes [24,25], skin [26], and cardiovascular tissues [27] as well as other biological tissues. The application of OCT in stomatology is gradually being explored, especially in early diagnosis, treatment, and prognosis monitoring of dental caries, periodontitis, and oral mucosal diseases [28,29,30,31,32,33,34,35].

In the present study, the OCT device was used to scan and image the whole process of enamel white spot lesions of artificial rabbit teeth restored with resin infiltrant. The results were compared with a stereoscopic microscope, SEM, and HE staining at the same time to investigate the feasibility of the OCT device to monitor and guide the resin infiltrant restoration of WSLs in real-time.

## 2. Materials and Methods

### 2.1. Animals

All animal procedures were performed in accordance with the Guidelines for Institutional Animal Care and Use Committee and approved by the Guangdong quality supervision inspection station for healthcare devices (No. 2020102001, approved on 20 October 2020). Seven healthy male New Zealand rabbits (3 to 4 months old) with similar weights were purchased from Guangdong Medical Laboratory Animal Center. All rabbits had no oral disease, and the shapes of maxillary incisors were complete without white spots on the surfaces. Before experiments, the animals were housed in standard plastic cages and maintained on a 12 h light-dark cycle at an ambient temperature of 23–24 °C, and received routine feeding for 1 week.

### 2.2. Preparatory Steps

The experimental rabbits were randomly numbered from #1 to #7. Before the experiment, all rabbits fasted for 12 h. The rabbits were anesthetized by intravenous administration of pentobarbital sodium at a dose of 30 mg/Kg. The rabbits were fixed to expose their maxillary incisors.

The following steps were carried out in order. Step 1: the surfaces of maxillary incisors were clean only with a brush and pure water. Step 2: 37% phosphate etching agent (Guanya, Wuhan, China) was evenly applied to the labial surfaces of maxillary incisors. After static placement of 15 min, the surfaces were washed with flowing water for 30 s and were dried with compressed air. Step 3: Icon-Etch (DMG, Hamburg, Germany) was smeared evenly on the labial surfaces of maxillary incisors for 30 s, rinsed with flowing water for 30 s, and dried with compressed air. Step 4: Icon-Dry (DMG, Hamburg, Germany) was smeared evenly on the labial surfaces of maxillary incisors and kept for 1 min, after which the surfaces were dried with compressed air. Step 5: Icon-Infiltrant (DMG, Hamburg, Germany) was smeared evenly on the labial surfaces of maxillary incisors for 1 min and light-cured with a curing light (Dentsply, York County, PA, USA) for 40 s.

Step 1 was performed on Rabbit #3. Steps 1 and 2 were performed on Rabbit #4. Steps 1, 2 and 3 were performed on Rabbit #5. Steps 1, 2, 3 and 4 were performed on Rabbit #6. Steps 1, 2, 3, 4 and 5 were performed on Rabbits #1, #2 and #7.

### 2.3. OCT Scanning Imaging and SM Imaging

**Figure 1:**
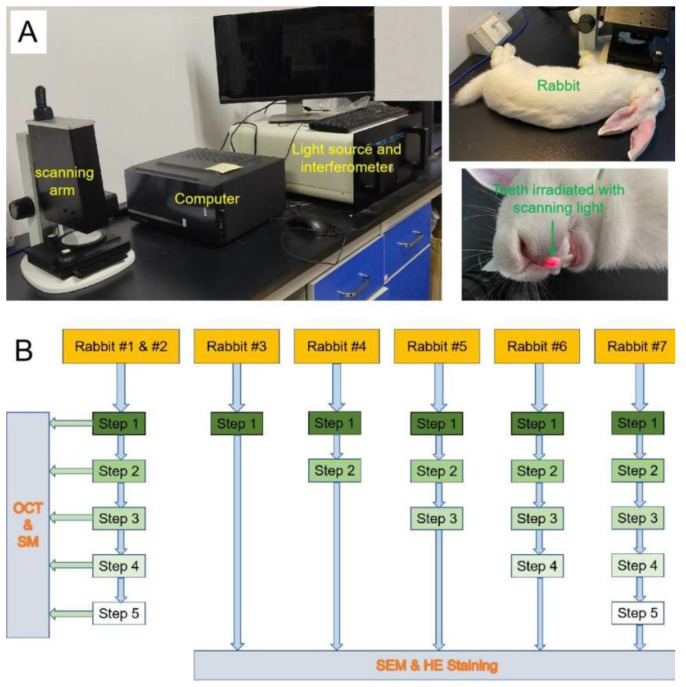
(**A**) The OCT system and a fixed rabbit, which teeth was irradiated with scanning light. (**B**) The schematic diagram of the protocol of this study.

Immediately after every preparatory step, the specimens of Rabbits #1 and #2 were scanned by an OCT system (as it was shown in Figure 1A) in our medical imaging innovation laboratory that was described in the previous study [23] and was observed and recorded under an SM (Leica, Wetzlar, Germany) at 35 magnifications. The OCT system used 840 nm central wavelength (λ), 40 nm bandwidth (Δλ), 5 MW power (P), and 7.24 μm axial resolution. In addition, the imaging depth of OCT in the teeth was over 400 μm. The OCT scanning probe was placed directly above the rabbit maxillary incisor so that the scanning optical path of the scanning optical fiber probe was perpendicular to the labial surface of the incisor, and the surface was scanned from tooth crown to tooth-root along the direction of the long axis of the tooth. The width and length of the scanning image were both 5 mm. The experimental protocol of this study was shown in Figure 1B.

### 2.4. SEM Imaging

After every preparatory step, the Rabbits #3, #4, #5, #6, and #7 were killed by injecting excessive anesthetics into their ear veins, and the maxillary incisors were completely removed and fixed in 4% neutral formaldehyde for 3 days. Their left incisors were used for scanning electron microscopy. After routine fixation, drying, and gold spraying, the teeth were observed using SEM (accelerated voltage is 15 KV) (Hitachi, Tokyo, Japan), and the images were recorded at 200, 500, 1000, 2000, and 3000 magnifications.

### 2.5. Hematoxylin and Eosin Staining

The extracted right incisors of Rabbits #3, #4, #5, #6 and #7 were immersed in an EDTA decalcifying solution (Sangon Biotech, Shanghai, China) [36]. After rinsing for 5 min with running water, the samples were dehydrated and embedded in paraffin (Xiya Reagent, Linyi, Shandong, China). Then the embedded sample was cut into sections with a thickness of 4 μm. After drying, the samples were dewaxed with xylene (Xiya Reagent, Linyi, Shandong, China), dehydrated with gradient ethanol (Xiya Reagent, Linyi, Shandong, China), rinsed with distilled water, stained with hematoxylin (Xiya Reagent, Linyi, Shandong, China) and eosin (Xiya Reagent, Linyi, Shandong, China), sealed with neutral resin (Solarbio, Beijing, China), and finally observed under a microscope (Leica, Wetzlar, Germany) and photographed.

### 2.6. Statistical Analysis

The OCT images were analyzed to obtain the light intensity of each pixel on the OCT cross-sectional image by MATLAB 2020b (The MathWorks Inc., Natick, MA, USA). All researchers discussed and determined the data acquisition standards and methods of analysis to ensure data uniformity. The two researchers were familiar with the standard and method through training, and conducted standard consistency test. The kappa value obtained was greater than 0.8. One of the researchers completed data collection and analysis independently, and another researcher verified it. Twenty locations on each OCT image of Rabbits #1 and #2 were randomly selected, and the light signal reflection/scattering bright band thickness of the enamel layer and dentin layer of each tooth was measured according to the change of light intensity. Baseline calibration was performed on each surface line of the selected locations, and the surface roughness of the sample was measured according to the arithmetic mean of the absolute value of the distance between each point on the sample surface and the baseline [23]. The surface roughness and the differences of changes in the light signal reflection/scattering bright band thickness of the enamel layer and dentin layer were subjected to one-way analysis of variance (ANOVA) followed by the Bonferroni post hoc test for multiple comparisons at a 5% significance level. *P* < 0.05 was considered statistically significant. Quantitative data were expressed as mean ± standard deviation. Statistical analyses were performed using Statistics 25 (SPSS Inc., IBM Company, Chicago, IL, USA). GraphPad Prism 8 (GraphPad Software, San Diego, CA, USA) and Origin 2021 (OriginLab Corporation, Northampton, MA, USA) were used to draw graphs based on the data obtained.

## 3. Results

### 3.1. OCT Scanning Images

**Figure 2:**
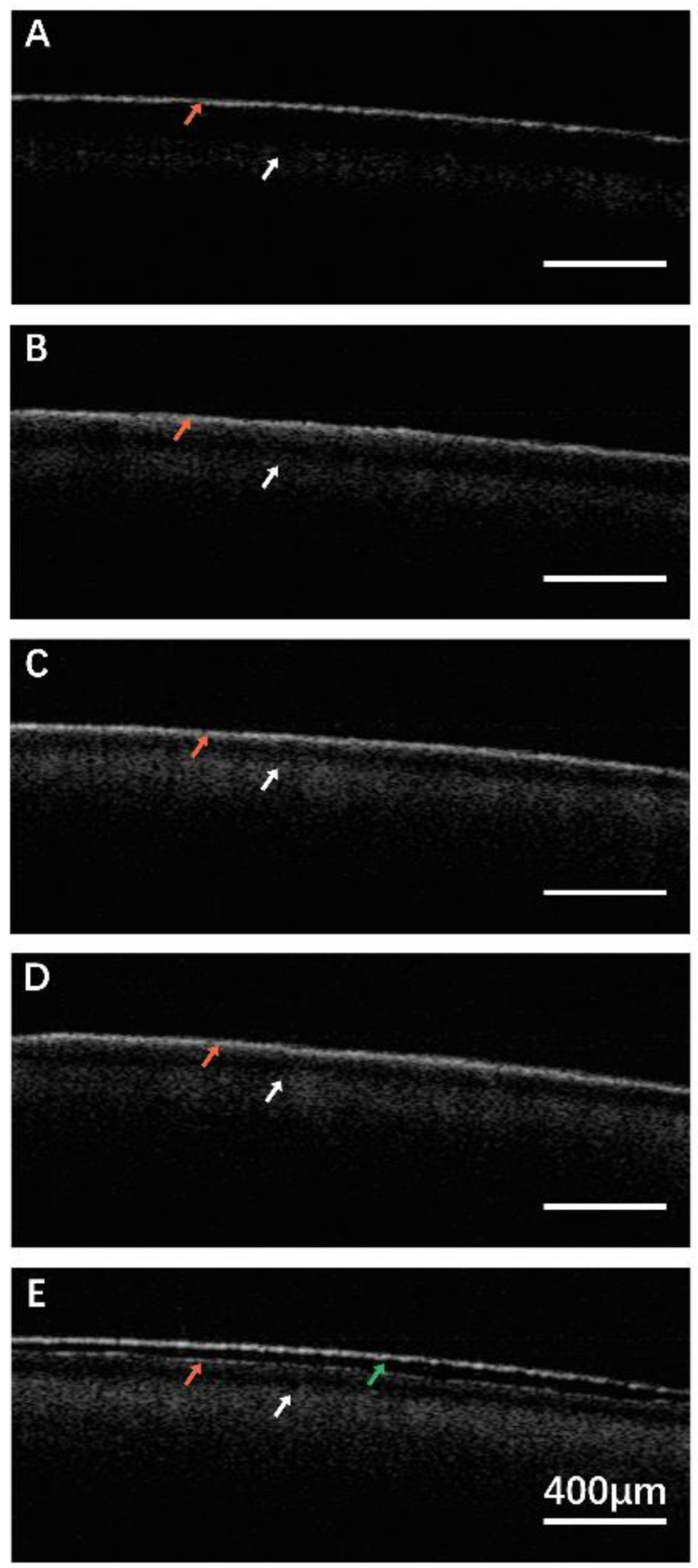
The OCT scanning images of each step. (**A**) Step 1: the surface of the incisor was clean; (**B**) Step 2: 37% phosphate etching agent was evenly applied to the incisor for 15 min, after which the surface was clean and dried; (**C**) Step 3: Icon-Etch was smeared evenly on the surface of incisor for 30 s, after which the surface was clean and dried; (**D**) Step 4: Icon-Dry was smeared evenly on the surface of incisor and kept for 1 min, after which the surface was dried; (**E**) Step 5: Icon-Infiltrant was smeared evenly on the surface of maxillary incisor for 1 min and light-cured for 40 s. Red arrow: the surface of the enamel. White arrow: enamel-dentinal junction (EDJ). Green arrow: resin layer. Scale bar is 400 μm.

In Step 1, a smooth light signal reflection bright band was observed on the surface of the enamel, without obvious scattering on the deep enamel and dentin (Figure 2A). In Step 2, the boundary between the reflection bright band of enamel surface light signal and the scattering bright band of lower deep enamel light signal was not obvious. Some superficial dentin showed the obvious scattering bright band, and the EDJ was blurred labeled by the white arrow (Figure 2B). In Step 3 (Figure 2C) and Step 4 (Figure 2D), there was no obvious boundary between surface light signal reflection bright band and lower deep enamel light signal scattering bright band, but the dentin scattering bright band was more obvious than Group 1, and the EDJ was clearly labeled. In Step 5 (Figure 2E), a light signal reflection bright band was seen on the surface of the resin layer. No obvious scattering was observed in the deep resin labeled by the green arrow. A light signal reflection light band near the surface of the enamel and a scattered light band in the superficial dentin, as well as the EDJ, were seen.

### 3.2. Intensity Profile of OCT Images

**Figure 3:**
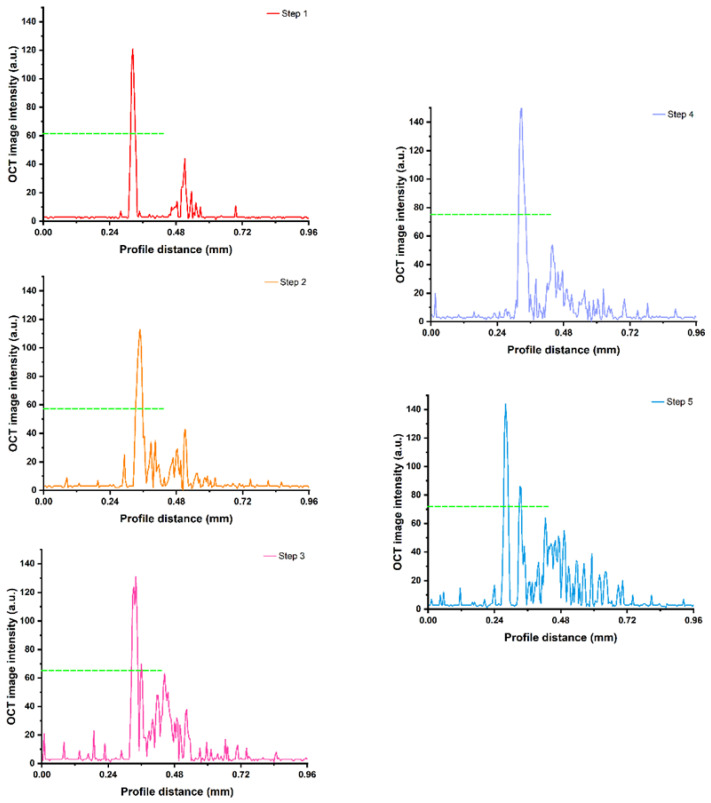
The OCT image intensity with profile distance in Steps 1, 2, 3, 4 and 5. Dashed line: the half maximum of each step. The half maximum OCT image intensity in Step 4 was the maximum, followed by Step 5, Step 3, Step 1, and Step 2.

An image intensity profile was extracted from the OCT image of the rabbit tooth for quantitative analysis of the enamel-dentinal junction and the resin layer (Figure 3). The half maximum OCT image intensity in Step 4 was the maximum, followed by Step 5, Step 3, Step 1, and Step 2.

### 3.3. SM Images, SEM Images, and Histology

**Figure 4:**
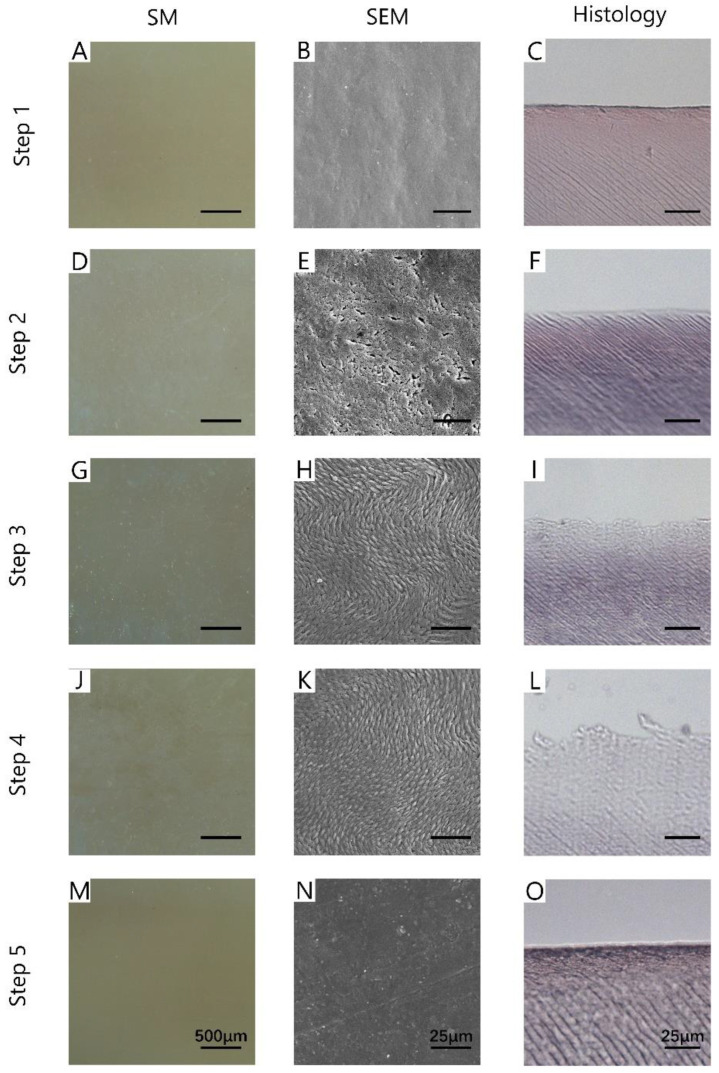
The images of SM (**A**,**D**,**G**,**J**,**M**), SEM (**B**,**E**,**H**,**K**,**N**), and histology (**C**,**F**,**I**,**L**,**O**) of each step. Step 1: the surface of the incisor was clean; Step 2: 37% phosphate etching agent was evenly applied to the incisor for 15 min, after which the surface was clean and dried; Step 3: Icon-Etch was smeared evenly on the surface of incisor for 30 s, after which the surface was clean and dried; Step 4: Icon-Dry was smeared evenly on the surface of incisor and kept for 1 min, after which the surface was dried; Step 5: Icon-Infiltrant was smeared evenly on the surface of maxillary incisor for 1 min and light-cured for 40 s. Scale bar of SM is 500 μm; scale bar of SEM is 25 μm; scale bar of histology is 25 μm.

For SM images, the enamel on the surface of the teeth in Step 1 was smooth and shiny (Figure 4A). A large area of dense white cloud-like spots was observed on the tooth surface in Step 2 (Figure 4D). The surface reflectivity of Step 3 (Figure 4G) and Step 4 (Figure 4J) was weak, and the dense white stripe structures were observed. In Step 5 (Figure 4M), the surface color was even, and no white spots were observed.

The second column of Figure 4 showed the SEM images of each step. As shown in Figure 4B, the surface in Step 1 was flat and dense, and no cracks were found. The tooth surface in Step 2 (Figure 4E) was full of pits. As for Step 3 (Figure 4H) and Step 4 (Figure 4K), the surface showed orderly fusiform structures, which were the basic structures of enamel. In Step 5 (Figure 4N), the tooth surface was dense and flat, with a discontinuous protuberance or depression area.

As for histology, the enamel surface in Step 1 (Figure 4C) was complete and the structure was compact. In Step 2 (Figure 4F), the enamel surface began to become incomplete, the outermost structure was sparse, and transparent changes appeared. After the treatment with Icon-Etch (Step 3, Figure 4I) and Icon-Dry (Step 4, Figure 4L), the enamel surface became more uneven, the outermost structure became sparser, and the transparent changes were more obvious. After the treatment with Icon-Infiltrant (Step 5, Figure 4O), the enamel surface of the sample was complete, and the tooth structure was compact as they were in Step 1.

### 3.4. The Thickness of Bright Area of Enamel and Dentin Layer in OCT Images

**Figure 5:**
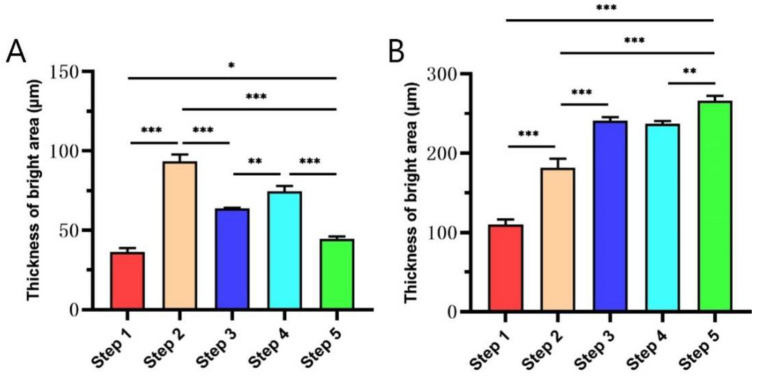
The thickness of the bright area of enamel (**A**) and dentin (**B**) in OCT images changed greatly in each step. * *p* < 0.05, ** *p* < 0.01, and *** *p* < 0.001.

Figure 5 illustrates that the comparison of the thickness of the bright area of enamel and dentin in OCT images changed greatly in each step. There was a significant difference between the steps (*p* < 0.05) (Figure 5A). Among these, the thickness of the bright area of the enamel layer in Step 2 is the largest, indicating that the enamel in Step 2 has the strongest scattering effect on the detection light, suggesting that there are more pores in the enamel layer in this step than in other steps. The thickness of the bright area of the enamel layer in Step 1 was the thinnest, suggesting that the pores in the enamel layer were less than those in other steps. The thickness of the bright area of the enamel layer between Step 1 and Step 5 has a significant difference (*p* = 0.043), suggesting that the pores of the enamel white spots were reduced after being repaired by resin infiltrant, but the normal enamel could not be completely restored.

The comparison of the thickness of the strong signal bright band of the dentin layer among the groups was shown in Figure 5B. The thickness of the bright area of the dentin layer in Step 1 was thinner than that in the other steps, indicating that the light passing through the enamel-dentinal junction into the dentin in Step 1 was the least, suggesting that the light was consumed more when penetrating the outer enamel. The thickness of the bright area of the dentin layer in Step 2 was thicker than that in Step 1 (*p* < 0.001), suggesting that the light consumption of the enamel layer in this group is reduced, which may be related to the decrease of mineral composition and the increase of pores in the demineralized enamel layer. In the process of resin infiltrant restoration, the thickness of the strong signal bright band of the dentin layer increased, suggesting that the resin infiltrant consumes less light than the air in the pores.

### 3.5. Enamel Surface Roughness

**Figure 6:**
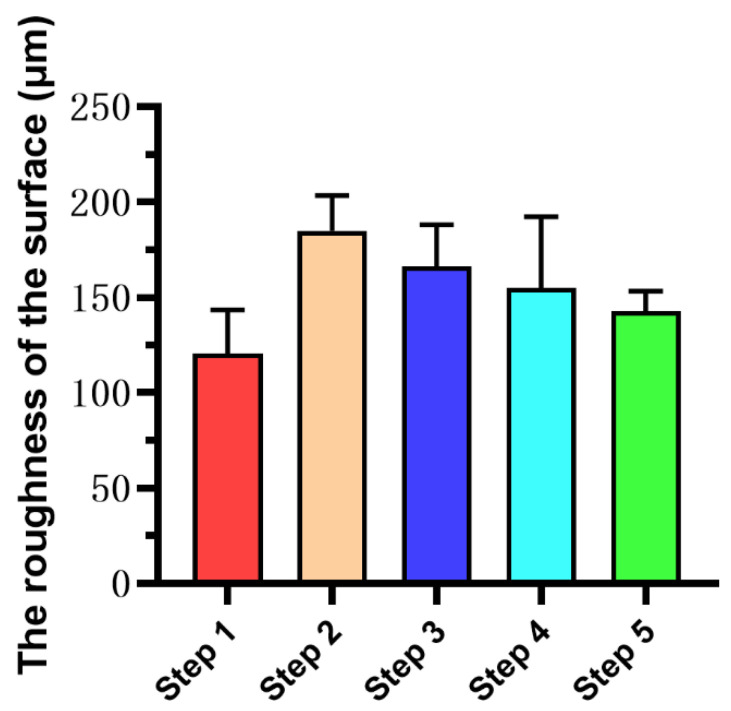
The roughness of the surface of teeth in each step. There was no statistical difference between steps. The surface roughness of Step 5 was similar to that of Step 1. The surface roughness of the samples of Step 2 increased due to demineralization, and the surface roughness of the samples was decreased after treatment with Icon-Etch (Step 3) and Icon-Dry (Step 4).

Figure 6 illustrates the results of enamel surface roughness calculated by OCT images at each step. Although there is no statistical difference in the surface roughness among the steps (*p* > 0.05), it can still be observed from Figure 6 that the surface roughness of Step 5 was similar to that of Step 1, which is the smoothest among the five groups. The surface roughness of the samples of Step 2 increased due to demineralization, and the surface roughness of the samples was decreased after treatment with Icon-Etch (Step 3) and Icon-Dry (Step 4).

## 4. Discussion

In the present study, 37% phosphoric acid etching was used to successfully establish the enamel white spot lesions model on rabbit maxillary incisors, which is similar to humans in the general structure of enamel and dentin [37], and resin infiltrant was used to repair the white spot lesions. OCT was used for real-time imaging before, after demineralization, and during operation of resin infiltrant. The results were compared with a stereoscopic microscope, scanning electron microscope, and HE staining.

Considering the existence of heartbeat, respiration, and muscle activity, this study simulated the interference of body activity on the imaging process during OCT imaging in living animals, so as to ensure that the research results were closer to the actual situation. The results prove that, based on the intensity profile of the OCT image, a quantitative evaluation of the enamel white spot lesions during the repairing process is feasible. As it was shown in Figure 2A, the surface of normal enamel is smooth and has a solid ability to reflect light, which shows a bright area with a strong reflection of the surface light signal in OCT images. White spots can be observed on the surface of demineralized samples, of which pore-like depressions can be observed on the surface of SEM images. In the OCT image, this part of the enamel has a solid ability to scatter light, showing a significantly thicker scattering area than that of the natural surface (*p* < 0.001). After treatment with Icon-Etch and Icon-Dry, the morphology of enamel rods was exposed (as they were shown in Figure 4H,K), showing bright areas of enamel light signal scattering in OCT. Treatment with Icon-Infiltrant restored the surface to be flat and smooth, while OCT also recovered the strongly reflective bright areas of the surface light signal, and the thickness of enamel light signal bright areas was smaller than that of demineralized enamel (Step 2) (*p* < 0.001), and still thickened compared to the natural surface (*p* = 0.043). Because the light scattering intensity of enamel is related to the number of micropores in the enamel [21], the decreased light reflectivity of demineralized enamel after treatment with resin infiltrant indicates that the number of micropores in the demineralized enamel reduced, consisting with the results of pathological HE staining. SEM images showed that the surface of the teeth changed significantly before and after demineralization, during and after restoration with resin infiltrant. At the same time, OCT can further calculate the data to obtain the specific situation of the surface roughness of the sample. The above experimental results show that OCT imaging can accurately and directly reflect the changes of tissue structure and surface morphology of tooth enamel layer before and after demineralization, and during and after restoration with resin infiltrant.

The present study illustrates that OCT brings a new way to evaluate the effect of resin infiltrant before and after repair. Seeliger et al. [38] used OCT to investigate the effect of the manner of enamel etching on its thickness before and after orthodontic treatment, and concluded that the effects of the classic etching method and the self-etching method on the enamel are similar. Ravichandran et al. [39] and Wijesinghe et al. [34] demonstrated the potential of OCT to assess enamel loss or enamel thickness of human teeth. OCT imaging before restoration of the demineralized area can accurately show the demineralization depth of the lesion area (Figure 2A,B). When the depth of demineralization exceeds the conventional penetration depth of resin infiltrant (20–465 μm) [40], the clinicians may consider whether it is necessary to use microabrasion technique to remove some of the demineralized enamel and reduce the thickness of the demineralized layer prior to the restoration with resin infiltrant [41]. Because of the high imaging resolution at the micron level of OCT, there is a significant difference in light scattering and reflection signals between resin infiltrant and dental tissue on OCT images, which makes it possible to clearly distinguish the two. OCT can penetrate the surface of dental tissue and show the image changes of deep tissue in the range of 2‒3 mm, which is more abundant than a visual diagnosis that can only obtain surface information. In addition, it works without ionizing radiation [42], avoiding the adverse effects of ionizing radiation on the human body, and is more in line with the minimally invasive concept pursued by modern medicine [43]. A study by Shimada et al. [44] showed that OCT imaging is more sensitive than visual inspection using conventional imaging diagnosis tools to diagnose occlusal caries.

SEM and HE staining can obtain the information of enamel white spot lesions before and after the restoration of resin infiltrant. However, there is irreversible damage to the tissue and the operation is complicated and time-consuming, so it is not operable in clinics. OCT is a high-resolution, non-destructive biological tissue optical imaging technology [32], which can make a reliable imaging diagnosis of the lesion without causing damage to the tissue. Vardar et al. [45] used both SEM and OCT equipment to assess the neointimal tissue, and the results showed that areas of neointimal tissue quantified by OCT equipment strongly correlate with SEM evaluation (*p* < 0.001).

In summary, OCT technology can be used for the whole process of resin infiltrant repair of enamel white spot lesions, with the advantages of non-invasion, high resolution, no ionizing radiation, and real-time in-vivo tissue imaging. It can effectively guide clinical practice and can be used as an imaging tool to evaluate the process and effect of resin infiltrant. Coupled with its significant advantages in monitoring tooth demineralization, OCT has great potential in monitoring tooth caries and restoration. However, OCT devices still have some shortcomings, such as poor flexibility of the scanning probe, heavy equipment, and shallow imaging depth, which limit its clinical application and require continuous improvement. This study discussed the morphological changes and OCT images of enamel white spot lesions before and after restoration with resin infiltrant. The prospect of the practical application of OCT technology in the restoration of enamel white spot lesions with resin infiltrant still needs further study. In this study, the central wavelength of the OCT system is 840 nm. Due to the influence of refraction and scattering, the longer central wavelength of the OCT system allows deeper penetration in a certain range [33,46,47,48]. In the future, the OCT system with a longer central wavelength will be studied.
